# Evaluation of a Water-Based Medium-Expansion Foam Depopulation Method in Suckling and Finisher Pigs

**DOI:** 10.3390/ani12081041

**Published:** 2022-04-16

**Authors:** Justin D. Kieffer, Magnus R. Campler, Ting-Yu Cheng, Andréia G. Arruda, Brad Youngblood, Steven J. Moeller, Andrew S. Bowman

**Affiliations:** 1 Department of Animal Sciences, College of Food, Agricultural, and Environmental Sciences, The Ohio State University, Columbus, OH 43210, USA; kieffer.22@osu.edu (J.D.K.); moeller.29@gmail.com (S.J.M.); 2 Department of Veterinary Preventive Medicine, College of Veterinary Medicine, The Ohio State University, Columbus, OH 43210, USA; campler.1@osu.edu (M.R.C.); cheng.1784@osu.edu (T.-Y.C.); arruda.13@osu.edu (A.G.A.); youngblood.25@osu.edu (B.Y.)

**Keywords:** swine, suckling, finisher, water-based foam, disease, depopulation

## Abstract

**Simple Summary:**

The need for effective and efficient means of mass depopulation of swine is critical, given foreign animal diseases, natural disasters, and other hazards that threaten swine production. Mass depopulation methods that can be applied to swine at all ages under field conditions are important due to the varying sizes and differing handling methods across the stages of swine development. The American Veterinary Medical Association Guidelines for Mass Depopulation contain limited options for procedures under constrained circumstances that can be applied rapidly across all sizes of pigs. Previous work has shown that water-based foam can be as effective as carbon dioxide for adult swine depopulation, but without CO_2_ supply, expensive equipment, and potential human health hazards. The present study demonstrates that water-based foam is also effective for depopulating, in suckling (18 to 24 days) and finisher (63 to 100 days) pigs. When combined with previous findings, water-based foam is a depopulation method that can be applied rapidly and effectively to all ages in the swine production cycle.

**Abstract:**

The threat of foreign animal disease introduction through contaminated animal products, feed ingredients, and wildlife vectors have highlighted the need for additional approved methods for mass depopulation of swine under emergency scenarios, especially methods that can be applied to pigs across all production phases. The market disruption within the swine industry due to the SARS-CoV-2 pandemic has demonstrated this lack of preparation. The objective of this study was to validate water-based foam as a mass depopulation method for suckling (18 to 24 days of age) and finisher stage (63 to 100 days of age) pigs. Finisher pigs (*n* = 31, originally 32 but one finisher pig died prior to foaming), allocated as 9 triads and 1 set of 4 pigs, in 10 total replicates, and suckling pigs (*n* = 32), randomly allocated to two replicates, were completely covered in water-based medium-expansion foam for a 15-min dwell time in a bulk container. Container fill time for the trials were 6.5 ± 0.68 s and 5.3 ± 0.03 s for finisher and suckling pig replicates, respectively. Average (± SD) time for cessation of movement was 105 ± 39.1 s (s) for finisher pigs and 79.5 ± 10.5 s for suckling pigs. After completion of the 15-min dwell time in the foam, all pigs were confirmed dead upon removal from the container. The results from the present study suggest that the use of water-based foam can be an effective means of mass depopulation for suckling and finisher stage pigs, supporting previous research on the application to adult swine.

## 1. Introduction

The spread of African Swine Fever throughout Asia, Europe and now the Caribbean [1], and the impact of the SARS-CoV-2 pandemic on the US swine industry have demonstrated the need for further development of mass depopulation methods for swine. The use of the term “depopulation” in the context of this study refers to the definition of the word supplied in the 2019 AVMA Depopulation Guidelines: “The rapid destruction of a population of animals in response to urgent circumstances with as much consideration given to the welfare of the animals as practicable. Urgent circumstances may include emergency situations, such as the need for immediate disease control, or a response to a natural or human-made disasters” [2]. Depopulation methods within this definition include, but are not necessarily limited to, AVMA-approved methods of humane euthanasia and humane slaughter. The issue of implementing mass depopulation to swine in the event of a foreign animal disease incursion or harvest disruption due to labor availability is multifaceted. Given the multiple stages of production, housing differences (e.g., group pens vs. gestation crates vs. farrowing crates), space limitations, time constraints, and varying sizes and ages of swine, no currently available depopulation method can be considered a blanket solution. Thus, there is a need for a rapid, universal, and reliable swine depopulation methodology, which aims to be as humane as possible, while reducing the anxiety experienced by the animals prior to loss of consciousness. The current AVMA Guidelines for the Depopulation of Animals [2] list several physical (penetrating captive bolt, gunshot, non-penetrating captive bolt, electrocution, and blunt force trauma), one gaseous (CO_2_), and one chemical (injectable barbiturate overdose) method for mass depopulation. While these methods are AVMA approved [3] and work rapidly to initiate unconsciousness and insensibility, they are administered at the individual level (with the exception of CO_2_ and transport to slaughter) and are generally not practical on a mass scale, given safety concerns, mechanical wear and tear, risk of contagion, and human physical and mental strain. Moreover, not all of the physical methods are the best choice for all sizes of pigs [2]. For example, electrocution is not recommended for piglets under 4.5 kg, non-penetrating captive bolt use is only effective for suckling and nursery pigs, and gunshot/penetrating captive bolt are only appropriate for growing and adult swine, and not suitable for neonates and weanlings [2].

Ventilation shutdown (VSD/VSD+) is a mass depopulation method for swine, only recommended by the AVMA under constrained conditions [4]. This method has been utilized during the SARS-CoV-2 pandemic by producers to mass depopulate swine due to market disruptions in the slaughter phase of the industry. One case study [4] was generated during the mass depopulation of approximately 250,000 swine (nursery and finisher sizes) by VSD+ at a farm in Iowa, during the early months of the SARS-CoV-2 pandemic. The results indicated that this method was not able to reach 95% death rate in less than the one-hour limit for VSD+, per AVMA Depopulation Guidelines. The inability to reach targeted death rate within the desired timeframe occurred despite extensive modifications to the barn, including the addition of steam and heat in the absence of ventilation. Anothercase study documenting the impact of unintended ventilation failure in a swine facility in the UK reported 30 sow deaths with others in the facility surviving in temperatures of 35 °C for over 16 h [5]. The findings of these case studies identify the challenges of VSD+, particularly related to the noted extension of time to death (1 h or more) for a portion of the populations. There is a demonstrated need to identify depopulation methods in all sizes of swine that minimize pain and suffering.

Currently, the use of inhalants, such as carbon dioxide (CO_2_) and nitrogen (N_2_), may be a viable option for swine mass depopulation, as multiple animals, regardless of age, size, and weight, can be terminated simultaneously. However, there is concern regarding the aversiveness of gases when inhaled [6,7,8] and few depopulation studies using CO_2_ for swine have been undertaken in field conditions [9,10,11,12,13]. In addition, the use of CO_2_ requires the availability of large amounts of the gas, associated gas delivery equipment, including vaporizers, and airtight containers to apply and hold the gas and pigs. An alternative to gas inhalants is water-based foam, which is currently approved for use in the poultry industry [14,15,16]. The elimination of the gas component would minimize any hazardous risks for staff in case of equipment malfunction and reduce the risks associated with external gas shortages, delivery restrictions, and delays during a time of emergency. The pumps, hoses, nozzles, water tanks and foaming concentrate utilized in this study are readily available from industrial and firefighting supply vendors and complement the existing foaming supplies in the US Veterinary Stockpile. Foam can be applied to pigs in multiple types of containers, given that the containers are able to prevent foam from leaking out. In addition, water-based medium-expansion foam has recently been determined to be as effective as CO_2_ for depopulating cull-sows [17]. To investigate if water-based foam would have the same universal applicability as CO_2_, the objective of this study was to evaluate the efficacy of water-based foam as a depopulation agent in suckling and finisher stage pigs.

## 2. Materials and Methods

### 2.1. Ethics and Institutional Oversight

This study was completed in accordance with the animal use protocol 2020A00000036, which was approved by the Institutional Animal Care and Use Committee at The Ohio State University. A penetrating captive bolt device was available in the event of the need for supplemental euthanasia after completion of the depopulation method and animal removal from the container.

### 2.2. Animal Subjects

Thirty-two finisher stage pigs (average bodyweight 55.9 kg, range: 21 to 82 kg; age 63 to 100 days) and thirty-two suckling pigs (average bodyweight 3.2 kg, range: 1.8 to 10.4 kg; age 18 to 24 days) of mixed sex were obtained from The Ohio State University Swine Center and housed on-farm according to the Ag Guide [18] specifications until utilized in their respective trials.

Ten finishing stage and ten suckling pigs were selected and equipped with subcutaneous activity data loggers (DST-Centi-HRT, Star-Oddi, Garðabær, Iceland) approximately 24 h prior to depopulation. Pigs receiving the data loggers were placed under general anesthesia using Telazol^®^ (50 mg tiletamine, 50 mg zolazepam powder) reconstituted with 2.5 mL xylazine (250 mg total) and 2.5 mL ketamine (250 mg total) administered at 1 mL/27.2 kg of bodyweight intramuscularly in the rear leg. After confirmation of each pig achieving a surgical anesthetic plane, the pigs were placed in lateral recumbency and an area behind either the left or right triceps was blocked with 2% lidocaine and clipped and scrubbed aseptically with betadine soap and isopropyl alcohol. An approximately 2.5 cm incision was made, and space created subcutaneously for the logger via blunt dissection. The data loggers were approximately 50 mm in length and 15 mm in diameter. After device placement, the wound was closed with 35 mm (35 W) surgical staples. Pigs were monitored, and all recovered from anesthesia within 30 minutes (min). Finisher pigs fitted with an implanted monitor were individually housed following recovery and prior to depopulation to prevent injury to the implant site by other pigs. Suckling pigs were placed with their respective littermates following implantation. One finisher pig (not implanted) in an anesthetized replicate died due to unknown causes (no pathology noted on post-mortem examination) prior to foaming, leaving thirty-one finisher pigs for the study.

### 2.3. Field Trial Experiments

**Figure 1:**
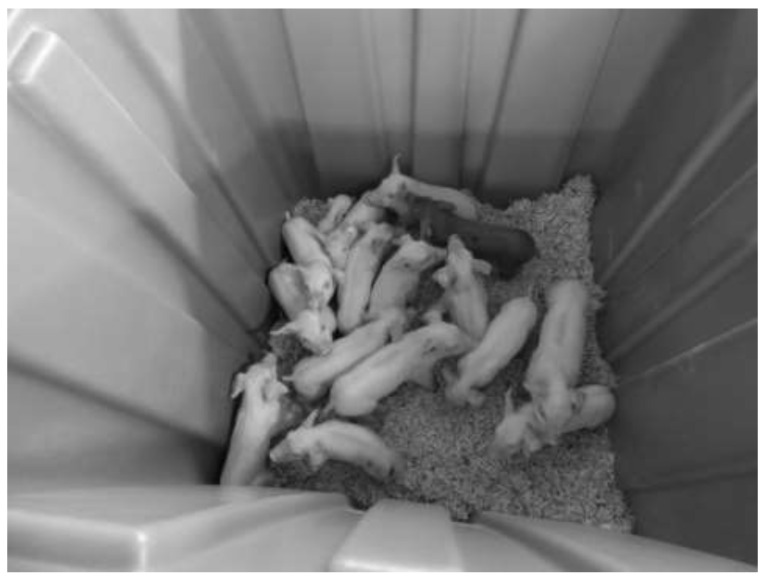
Conscious suckling piglets in a 1.46 m^3^ (1.12 m × 1.12 m × 1.14 m: length, width, and height) chamber with a layer of 15 cm pine shavings prior to depopulation.

A commercially available bulk polyethylene container (Uline, Inc., Pleasant Prairie, WI, USA) was used as the depopulation chamber, with a volume of 1.46 m^3^ (1.12 m × 1.12 m × 1.14 m: length, width, and height). The bottom of the chamber was filled to a depth of approximately 15 cm with pine shavings to provide footing for the pigs during the experiment (Figure 1). The number of pigs within a replicate was determined by, and did not exceed, transport stocking density recommendations for the size of pigs utilized in each trial [12].

In the finisher pig trial, the 31 pigs were assigned either to an anesthetized group (*n* = 15) or conscious group (*n* = 16) prior to the water-based foam depopulation. Anesthetized groups were utilized in the finisher pig trial to confirm that the foam would be lethal at the end of the 15-min dwell time, similar to the approach reported in a previous cull sow foam study [17]. Following confirmation of the efficacy of the foam in the anesthetized finisher pigs, the trial continued with the conscious groups of finisher pigs. The anesthetized group was split into 5 replicates of 3 pigs each, and the conscious group was divided into 4 replicates of 3 pigs and 1 replicate of 4 pigs. Mixed combinations of light- (21 to 23 kg), medium- (32 to 36 kg) and heavy-weight (45 to 55 kg) pigs were represented in each replicate. Further, 1 pig in 10 of 10 total finisher replicates was implanted with a subcutaneous data logger, with 3 lightweight, 4 medium-weight and 3 heavyweight pigs implanted. Unconscious pigs were anesthetized via the above-listed protocol. A surgical plane of anesthesia was confirmed for each pig by lack of corneal reflex, lack of response to noxious stimuli, and lateral recumbency. Once a surgical plane of anesthesia was confirmed, the pigs within a replicate were placed in the container and foam was applied.

**Figure 2:**
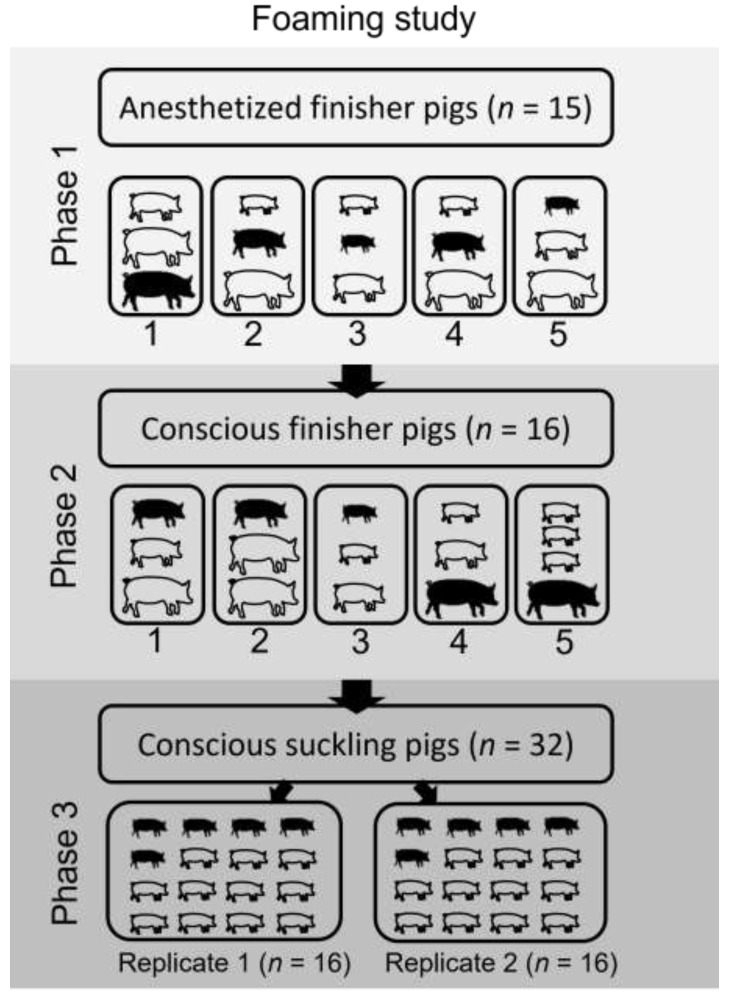
Chronological study phase progression for anesthetized finisher pigs (*n* = 15), conscious finisher pigs (*n* = 16) and conscious suckling pigs (*n* = 32). The number of replicates for each phase is broken down and demarked in black outline. The number of pigs and pig sizes within each finisher pig replicate correspond to pigs of each weight category (light = 21 to 23 kg; medium = 32 to 36 kg; heavy = 34 to 55 kg). The number of suckling pigs (mean weight = 3.2 ± 0.8 kg) only indicate the number of pigs used per replicate and size does not correspond to any above-mentioned finisher weight category. Pigs with an implanted activity logger are represented as outlined in black for each phase and replicate.

For the suckling pigs, the trial was completed with two replicates of 16 piglets per foaming event. Five piglets within each replicate were implanted with the subcutaneous data logger. All suckling pigs were depopulated from a conscious state. A diagram of the study design is presented in Figure 2.

### 2.4. Foam Production and Application

**Figure 3:**
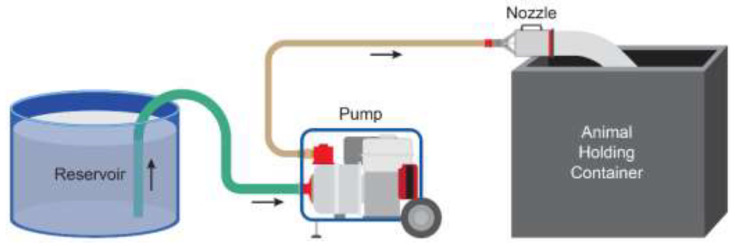
Schematic drawing of the equipment setup used for water-based foam depopulation. The foam concentrate and water are mixed to the desired concentraiton in the Reservoir. The water and foam concentrate solution is delivered to the medium-expansion foam handline nozzle via hoses connected to a gasoline-powered high-pressure water pump.

Foam was generated during the trials using PHOS-CHEK^®^ WD881 Class A foam concentrate (Perimeter Solutions, Rancho Cucamonga, CA, USA) added to an 1892 L water tank to create a 1% foam-water solution (Figure 3). The foam-water solution was pumped from the tank by a gasoline-powered water pump (Honda Power Equipment WH20, Alpharetta, GA, USA) via a 5.08 cm diameter suction hose connected to the pump inlet. The foam-water solution was delivered out of the pump in a 3.81 cm diameter firehose (15 m in length) to an aspirated foam nozzle (KR-M4, ANSUL, Marinette, WI, USA). The firehose was connected to the water pump via a 5.08 cm diameter brass female National Pipe Straight Hose Thread (NPSH) and a 3.81 cm diameter male national hose fire hose adapter (W.W. Grainger Inc., Lake Forest, IL, USA). One research team member operated the water pump while another operated the nozzle to fill the depopulation chamber. Both conscious and anesthetized pigs were placed manually in the chamber on top of the bed of shavings, and the chamber was then filled to the top with water-based foam. Top offs of foam were not required, as no foam breakdown was noticed during the 15-min dwell time. After completion of the 15-min immersion period, the container was dumped and the pigs were evaluated for confirmation of death based upon absence of corneal reflex, lateral recumbency and respirations.

### 2.5. Activity Measurement

The DST-Centi-HRT subcutaneous data logger uses tri-axial acceleration technology to measure activity as external acceleration (EA) forces deviating from the normal force of gravity (g, 9.8 m s^−2^) placed on the data logger in its resting state. Raw acceleration is measured on each of the three axes and later normalized to 1 g based on measurements for each axis during rotation. All data were downloaded from the data loggers using the associated Mercury software v5.99 (Star Oddi, Gardabaer, Iceland). The software algorithm calculates the EA as a vectorial sum of dynamic body acceleration in any plane (veDBA; reported by the software in mg), allowing visualization of EA and axis plane force over time, indicating the force and direction of movement for each time stamp. The data logger was pre-set to record EA every 15 s throughout the duration of the study.

Due to the inherent sensitivity of the data logger, and the constant effect of natural g-forces applied in the resting state, an activity baseline for each animal was established. The activity threshold was established as the upper threshold for typical outlier detection in asymmetric distributed data [19]. In brief, the threshold was calculated as the third quantile of EA plus 1.5-times of the difference between first and third EA quantiles, i.e., inter-quantile range (IQR). Thus, movements considered to be related to locomotion or conscious pig activity were determined by any EA measurements above the threshold. Individual and age-group levels of thresholds, based on EA during the 15-min foam immersion, were calculated for each pig and those from all conscious pigs in the same age group (finisher: *n* = 5; suckling: *n* = 10), respectively. Thereafter, the time of cessation of movement (COM) was determined as the next consecutive measurement time point (15 s after) of a plausible movement that had no above-threshold EA in the following 5 min. For example, if a pig had an EA above the threshold at the 2nd minute post foaming with no plausible movement until the 7th minute, the time of COM was at 2 min and 15 s. Note, no data were collected on anesthetized implanted pigs as they did not move. The data loggers used were capable of monitoring heart rate along with activity; however, due to an error in programming no heart rate information was collected. Knowledge of the programming failure was not known until data loggers were retrieved and data transferred following all trial activities. Therefore, activity monitoring was only recorded on 5 finisher pigs, one within each of 5 replicate foaming events.

### 2.6. Statistical Analysis

Statistical analysis was performed in R 4.0.4 (R Core Team, 2021). The times of COM based on the individual and age-group level of thresholds, were compared by the age of pigs (finisher versus suckling) using a non-parametric two-sample Wilcoxon rank-sum test. In addition, regardless of the age of pigs, the comparison between the time of COM determined by individual and age-group-level activity thresholds was conducted using a non-parametric paired sample Wilcoxon signed rank test. Exact *p*-values were computed for all comparisons given the small sample size.

## 3. Results

The average foam fill time for the bulk container was 6.5 s ± 0.68 (mean seconds ± standard deviation) and 5.3 s ± 0.03, for the suckling piglets and finishing pigs, respectively. Death was confirmed in all animals after 15 min of foam immersion. Of the five conscious finisher pigs implanted with subcutaneous data loggers, the mean time to cessation of movement (COM) was 105 s ± 43.7 and 84 s ± 27.2 for the individual and age-group level of threshold, respectively. Two replicates of five implanted suckling piglets showed a mean time to COM of 168 s ± 120.7 and 123 s ± 111.4, using the individual-level threshold, and a mean time to COM of 48 s ± 19.6 and 57 s ± 40.2 using the age-group-level threshold. Further descriptive statistics of pig weights and time to COM are summarized in Table 1.

No difference between time to COM was found between suckling and finisher pigs, either using individual (Wilcoxon rank-sum test, U = 23.5, *p* = 0.88) or age-group-level (Wilcoxon rank-sum test, U = 40.5, *p* = 0.05) activity threshold. However, regardless of the age of pigs, the individual threshold determined equal or longer time to COM than the age-group-level threshold calculated for an individual pig (Wilcoxon signed rank test, W = 92, *p* = 0.008).

## 4. Discussion

The application of water-based medium-expansion foam to suckling and finisher pigs for depopulation was successful in the present trials, with all pigs confirmed dead at the time of removal from the container. No statistically significant differences in time to cessation of movement (COM), determined by individual or age group level of activity threshold, were noted between finisher and suckling pigs. However, a longer time to COM was determined by the individual-level threshold when compared with the age-group-level threshold. The mean times to COM in finisher pigs (132 s and 63 s determined by individual and age-group-level thresholds) and suckling pigs (145.5 s and 52.5 s for both individual and age-group-level activity thresholds) were comparable to those observed in previous small (186 s) and large-scale trials (128 s), using cull sows [17]. To our knowledge, only one other study has reported use of a foam-based approach for euthanasia/stunning in pigs that were similar in size used in the present trials. Lindahl et al. (2020) compared nitrogen and air-filled foam as a method for stunning finisher pigs individually, in a specially built windowed chamber, reporting mean time to loss of posture of 57 s and mean time to last observed muscle contraction to be an additional 131 s [20]. Combined, total time to last visual evidence of muscle activity was 188s on average in ~27 kg finishing pigs [20]. It is important to note that the finisher and suckling pigs administered water-based foam in the present study were depopulated in groups of 3 (finishers) or 16 (suckling), whereas Lindahl et al. (2020) foamed pigs individually.

Our previous work, comparing the use of N_2_, CO_2_, compressed air foam, compressed nitrogen foam, and aspirated water-based foam, showed that aspirated foam was similar in efficacy to the use of CO_2_ in the depopulation of cull sows [17]. Mean time to cessation of movement in aspirated foam in small-scale cull sow trials was 186 s and in large-scale trials, 128 s [17]. These mean times to cessation of movement were similar to CO_2_ in our small-scale trials [1]. Carbon-dioxide is AVMA-approved and effective for euthanasia and mass depopulation of swine of all sizes, and a large-scale field trial indicated that CO_2_ can work as a mass depopulation agent for feeders and adult swine [10,21]. Our previous study demonstrated aspirated-water-based medium-expansion foam is as effective for depopulation of larger swine as CO_2_, both in small-scale trials and field settings, without the complications of CO_2_ production. Previous and present results support the efficacy of water-based foam as an option for depopulation in complement to currently available options.

The capability of a depopulation method to be rapidly applied across different sizes of swine in an emergency scenario is important. Such capability not only simplifies the tools, techniques and training needed for the process, but also increases the speed of depopulation if the method is applied at the group level instead of at the individual level (e.g., captive bolt or gunshot). The tools necessary for water-based foam depopulation that we employed in the present study, including firefighting foam concentrate, water pumps, firehose and foaming nozzles, are relatively inexpensive, readily available, and, in part, are present in the National Veterinary Stockpile. The foam design described avoids the need for gas equipment and potential challenge of gas availability in CO_2_ systems and also eliminates the need for large numbers of captive bolt guns or firearms and associated ammunition for individual depopulation, all of which may become scarce in a national depopulation scenario. The findings are clear that water-based foam leads to COM and death in swine of all sizes within 15 min following administration in group depopulation settings, as compared to the AVMA conditionally approved Ventilation Shutdown method [4,17]. Reports also describe that the impacts of disease outbreaks and concurrent depopulation activities are associated with negative emotional impacts on the personnel administering the methods [22,23]. The use of water-based foam may reduce the impact of animal depopulation on human caretakers, as the application of the technique in a controlled containment area and the rapid covering of the animal with foam provides a visual barrier to the process once the animals have been foamed. More investigation is needed to determine the use of water-based foam compared to other depopulation methods, especially as it relates to worker mental health.

Pigs in our study were placed by hand into bulk containers for foam depopulation; however, this was performed due to the experimental nature of this study. Finisher and suckling-sized swine used in the present trials could be placed in larger containers, trailers or other compartments with solid sides and flooring amenable to holding medium-expansion water-based foam. In our previous work, we used a modified rendering trailer to depopulate groups of sows [17], as did Kinsey et al. (2016) and Pepin et al. (2022), when investigating carbon dioxide euthanasia [12,13]. The use of mobile containers, such as trailers, may also facilitate movement and disposal of the animals post depopulation, which will likely depend on causative pathogen and movement restrictions that may be in place. The time-limiting step when using containers or trailers for group depopulation in our experience was the time needed to move the swine from their housing units into the depopulation containers; however, the movement time would likely mimic the time required to load swine for normal transfer or marketing, unless pig movement was impeded by health status.

The results of our study indicate that water-based foam is a feasible method for the depopulation of finisher and suckling swine. Similar to previous work with water-based foam in cull sows [17], the foam enters the respiratory tract, creating a mechanical hypoxia and subsequent death. Despite a nominal level of replication, the approach described led to data indicating 100% efficacy in achieving rapid loss of movement and assurance of death at a predefined 15-min endpoint. It is important to note that terminal studies should always aim to use the lowest number of animals possible to address specific objectives. In the present study, preliminary data were already in place, which allowed us confidence on the success of the trials. More research is needed to evaluate the use of water-based foam in larger groups of finisher and suckling pigs, and within differing containers. The pigs in the present study were evaluated under transport-approved stocking density and clear delineation of distance and range of movement. Our experience suggests that application of foaming requires constraint in linear movement capability to avoid the potential for piling/climbing that can occur if very large groups are allowed to move freely. In addition, further investigation is needed to identify the time to loss of consciousness following foam application with greater confidence, and fully identify the foam and length of dwell time needed within foam to guarantee non-recovery of the animals. Since the regular activity was not recorded in this study, a typical statistical approach for data outlier determination was utilized to calculate EA thresholds. Two thresholds based on EA measurements of 15-min immersion from individual animals and animals in the same age group were calculated to comprehensively estimate the time of COM. Earlier time of COM was determined by the age-group threshold, rather than individual, because the former was skewed by extremely high EA values in certain animals. That is, age-group thresholds were higher than individual thresholds, and could be biased toward extreme EA from highly active animals. Thus, individual thresholds were reported to provide conservative estimates for time of COM. It is worth noting that this study determined outlying EA readings as plausible animal activity, which was dependent on the EA measurement throughout the foam immersion and could not capture all actual movements. Although visualization was impractical for water-based foam, posture, heartbeat, blood pressure, pulse, respiratory rate, and brain waves were not monitored during the depopulation process because of logistic hurdles. Thus, future studies down this research line should establish activity baseline by implanting animals with bio-loggers in advance, and cross-verify the exact time of non-recovery, unconsciousness, and death with other vital signs.

## 5. Conclusions

The results of this study indicate that the use of water-based foam as a mass depopulation agent for swine of suckling and finisher sizes is effective. The cessation of movement data of smaller and younger swine in this study are similar to previously published work in cull sows, using medium-expansion foam for depopulation in small-scale trials and in large groups. Water-based foam is a depopulation method that can be applied to swine of all sizes, which simplifies the depopulation equipment, training, and readiness process.

## Figures and Tables

**Table 1:** Pig body-weight range or individual pig weight and time to cessation of movement (COM) post-foam initiation for conscious, logger-implanted finisher and suckling pigs evaluated in water-based foam depopulation trials.

Age (Day)	Replicate	Weight (kg)		Time to Cessation of Movement(s) *	
				Individual Threshold **	Age Group Threshold **
Finisher 63–100	N/A	21.3		45	45
		35.8		90	90
		31.8		120	120
		45.8		165	75
		51.3		105	90
			Mean ± standard deviation	105 ± 43.7	84 ± 27.2
Suckling 18–24	1	4.7		345	30
		2.8		60	60
		3.3		240	45
		2.1		90	30
		2.9		105	75
			Mean ± standard deviation	168 ± 120.7	48 ± 19.6
	2	3.8		30	30
		2.8		60	60
		4.3		60	60
		2.7		300	120
		2.5		165	15
			Mean ± standard deviation	123 ± 111.4	57 ± 40.2

N/A = Not applicable. * Time to cessation of movement: 15 s after an external acceleration (EA) measurement exceed individual or age-group threshold with EA staying below thresholds in the following 5 min. ** Individual and age-group activity thresholds calculated using the following formula: ${T=\mathrm{EA}}_{Q3}+1.5{\times (\mathrm{EA}}_{Q3}-{\mathrm{EA}}_{Q1})\;.$
where T indicates thresholds; EA_Q1_ and EA_Q3_ the first and third quantile of EA measurements throughout the 15-min water-based foam immersion. The individual threshold was calculated using EA of each pig while the age-group threshold used EA from all pigs in each age group (suckling/finisher).

## Data Availability

Data available upon request.
